# A decadal profile of RhD-negative blood donors in Chongqing, China: demographics, phenotypic diversity, and donation behavior

**DOI:** 10.3389/fpubh.2025.1684831

**Published:** 2026-01-14

**Authors:** Yubi Gan, Chengbing Xie, Bujin Liu, Hongren Chen, Weifei Qin

**Affiliations:** Chongqing Blood Center, Chongqing, China

**Keywords:** donation behavior, phenotypic diversity, public health policy, RhD-negative blood donation recruitment, RhD-negative whole blood donors

## Abstract

**Background:**

RhD-negative blood is a critical resource in transfusion medicine due to its rarity and clinical significance. This study provides the first comprehensive analysis of RhD-negative blood donors in Chongqing, China over a decade (2015–2024) to optimize scientific research evidence for optimizing strategies to recruit and retain RhD-negative whole blood donors.

**Methods:**

A retrospective cohort of 4,185 RhD-negative donors who donated whole blood to the Chongqing Blood Center from 2015 to 2024 was analyzed. Variables included age, gender, occupation, education level, Rh phenotypes, ABO blood groups, and donation frequency were collected and analyzed to identify factors influencing whole blood donation.

**Results:**

In this study, the RhD-negative whole blood donors mainly consisted of donors who had donated only once, with an overall male-to-female ratio of 1.01:1. One-time donors were predominantly students (33.2%) and young adults (49.2% aged 18–25). Active donors correlated with higher education (university degree: 38.6%). The ABO blood group distribution was O > A > B > AB and dominant Rh phenotype was ccdee (50.7%), with rare variants (e.g., CCdEE) absent.

**Conclusion:**

This study highlights the need for tailored donor retention strategies and dynamic inventory management for rare subtypes. The findings outline a roadmap for blood centers to address RhD-negative blood shortages and enhance transfusion safety.

## Introduction

According to the International Society of Blood Transfusion, there are currently 48 recognized blood group systems encompassing more than 360 red cell antigens (as of May 2025) (1). The Rh blood group system is the most complex of all blood group systems (2). The D (RH1) antigen is the most immunogenic and clinically significant antigen, which directly affects the hemolytic transfusion reaction and hemolytic diseases of fetuses and newborns (2, 3). The distribution of RhD-negativity in China's Han population is very different from that of the European and American populations, with the frequency of RhD-negative phenotypes ranging from about 15–17 in the European and American populations (Caucasian), and only about 0.3 in China's Han population (4, 5). RhD-negative erythrocytes are a scarce resource in China, and the collection and supply of RhD-negative blood is often very difficult to meet the need for clinical blood (6, 7).

Despite its clinical urgency, few studies have systematically analyzed blood donation patterns, demographic characteristics, and information on the distribution of ABO and Rh blood groups among RhD-negative blood donors. This study bridges this gap by examining 10-year data (2015–2024) from Chongqing China. By retrospectively analyzing the population characteristics of RhD-negative blood donors in Chongqing during this period, our findings would provide a factual data foundation for formulate precise recruitment strategies and retention measures to optimize inventory management. Ultimately, this research aims to provide a scientific basis for establish a stable pool of RhD-negative blood donors and enhance preparedness for public health emergencies in Chongqing.

## Materials and methods

### Study participants

Data of 4,185 RhD-negative blood donors were collected at the Chongqing Blood Center. All donors met the health examination requirements for blood donation and successfully donated whole blood.

Test tube saline method for RhD and RhC,c,E,e detection. When RhD is negative by the test tube saline method, further RhD testing is conducted using the anti-human globulin method.

### Methods

This study was conducted at the Chongqing Blood Center, the institution responsible for blood collection and supply within the central urban area of Chongqing. Blood donor data were extracted from the BMIS (Business Management Information System, BMIS) system and all donors were voluntary non-remunerated donors according to the national and local blood donation policy in China. All donations were made voluntarily without direct financial compensation and were intended for the general blood supply rather than for a specific, pre-identified recipient. For the purpose of this study, donors were categorized based on their donation frequency. Those who donated blood only once (=1), regardless of the donation center or timing, were classified as one-time donors; those who had donated blood at least 2 times (≥2) and had donated at least once within the 12 months preceding the study were categorized as active donors; and those who had a history of blood donation who donated at least twice (≥2), but not in the last 12 months, were classified as the lapsed donors. The information related to RhD-negative blood donors' donation was categorized and compared retrospectively according to gender, age, education level, occupation, donation frequency, ABO blood group and Rh subtypes (ISBT classification). The correlation between these group characteristics and RhD-negative blood donation was analyzed. It is important to note that the information analyzed in this study were based on the donor's status at their first registration in BMIS system.

### Statistical analysis

We used Excel 2019 (Microsoft Corporation, Redmond, WA, USA) and SPSS (ver. 26.0, IBM, Chicago, Illinois) statistical software for data analysis. Count data were presented as rates or constituent ratios, and the χ^2^ test was used. *P* < 0.05 indicated that the difference was statistically significant.

## Results

### Demographic profile of RhD-negative whole blood donors according to donation frequency

During the study, we conducted a retrospective analysis of the data on RhD-negative whole blood donors over the 10-year period from 2015 to 2024. The demographic characteristics of the 4,185 RhD-negative whole blood donors according to donation frequency have been summarized in Table 1. In general, one-time donors represent the largest group (59.0%, 2,467/4,185). The proportion of both lapsed donors (33.6%, 1,407/4,185) and active donors (7.4%, 311/4,182) decreases as donation frequency rises, with active donors constituting the smallest share.

**Table 1:** Demographic distribution of RhD-negative blood donor cohorts.

**Characteristic**	**One-time donors number (%)**	**Lapsed donors number (%)**	**Active donors number (%)**	**χ^2^**	***P***
**Gender**					
Male	1,293 (52.4)	656 (46.6)	154 (49.5)	12.078	0.002
Female	1,174 (47.6)	751 (53.4)	157 (50.5)		
**Age group (years)**					
18–25	1,214 (49.2)	545 (38.7)	48 (15.4)	178.884	< 0.001
26–35	638 (25.9)	397 (28.2)	91 (29.3)		
36–45	348 (14.1)	256 (18.2)	90 (28.9)		
46–60	267 (10.8)	209 (14.9)	82 (26.4)		
**Education level**					
Junior high school and below	313 (12.7)	202 (14.4)	52 (16.7)	115.929	< 0.001
Senior high school	419 (17.0)	273 (19.4)	54 (17.4)		
Junior college	650 (26.3)	374 (26.6)	56 (18.0)		
Undergraduate and above	671 (27.2)	470 (33.4)	120 (38.6)		
Other	414 (16.8)	88 (6.3)	29 (9.3)		
**Occupation**					
Student	819 (33.2)	399 (28.4)	30 (9.6)	140.990	< 0.001
Peasant	101 (4.1)	54 (3.8)	15 (4.8)		
Civil servant	39 (1.6)	25 (1.8)	11 (3.5)		
Soldier	24 (1.0)	9 (0.6)	2 (0.6)		
Medical staff	69 (2.8)	55 (3.9)	29 (9.3)		
Factory worker	153 (6.2)	69 (4.9)	15 (4.8)		
Teacher	50 (2.0)	28 (2.0)	9 (2.9)		
Office staff	218 (8.8)	166 (11.8)	42 (13.5)		
Liberal professions	209 (8.5)	115 (8.2)	10 (3.2)		
Other	785 (31.8)	487 (34.6)	148 (47.6)		

One-time donors exhibited male predominance (52.4%), while lapsed donors had higher female representation (53.4%). Active donors showed balanced gender distribution (male: 49.5%, female: 50.5%, *p* = 0.002). One-time donors were younger than the other donor groups with 49.2% of donors being 18–25 years at the time of donating compared with 38.7% for lapsed donors and 15.4% for active donors, respectively (*p* < 0.001). Instead, active donors concentrated in older age brackets, with 28.9% aged 36–45 years and 26.4% over 46 years (Table 1).

Significant associations were observed between donation status and both educational attainment (*p* < 0.001) and occupation (*p* < 0.001) among RhD-negative whole blood donors. The educational level demonstrated a clear progression with increased donor commitment. Undergraduate degrees and above became increasingly prevalent, rising from 27.2% in one-time donors to 33.4% in lapsed donors, and reaching 38.6% among active donors, the largest proportion for any educational category within this group. Occupational distribution also varied markedly. Students constituted the largest single occupational group among one-time donors (33.2%) but represented only 9.6% of active donors. Office staff representation also increased with donation commitment (one-time: 8.8%, lapsed: 11.8%, active: 13.5%). Conversely, liberal professions decreased from 8.5% in one-time donors to 3.2% in active donors (Table 1).

### RhCcEe phenotype and ABO group distribution

Blood group analysis of 4,182 donors established O as the predominant ABO type (33.8%), followed by A (32.0%), B (24.4%), and AB (9.8%). A consistent hierarchical pattern of genotype distribution was evident across all ABO groups, with ccdee > Ccdee > CCdee collectively constituting over 94% of each subgroup.

The RhCcEe ccee phenotype dominated all ABO groups, accounting for 51.9% of group A and 49.7% of group O. Ccee demonstrated consistent prevalence across blood types (36.4–37.9%), while rare phenotypes (CCEe, CcEE, ccEE) collectively occurred in < 0.1% of the cohort, with ccEE detected only in single cases within A and B groups. Table 2 shows the prevalence of various Rh phenotypes and ABO group distribution in this study.

**Table 2:** ABO-RhCcEe phenotype distribution.

**Phenotype**	**A number (%)**	**B number (%)**	**0 number (%)**	**Ab number (%)**	**Total number (%)**
CCdEe	1 (0.1)	0 (0.0)	1 (0.1)	0 (0.0)	2 (0.0)
CCdee	80 (6.0)	78 (7.7)	123 (8.7)	31 (7.6)	312 (7.5)
CcdEE	1 (0.1)	0 (0.0)	0 (0.0)	0 (0.0)	1 (0.0)
CcdEe	23 (1.7)	10 (1.0)	20 (1.4)	4 (1.0)	57 (1.4)
Ccdee	494 (36.9)	386 (37.9)	514 (36.4)	151 (36.9)	1,545 (36.9)
ccdEE	1 (0.1)	1 (0.1)	0 (0.0)	0 (0.0)	2 (0.0)
ccdEe	44 (3.3)	25 (2.5)	53 (3.7)	20 (4.9)	142 (3.4)
ccdee	696 (51.9)	519 (50.9)	703 (49.7)	203 (49.6)	2,121 (50.7)
CCdEE	0 (0.0)	0 (0.0)	0 (0.0)	0 (0.0)	0 (0.0)
Total	1,340 (32.0)	1,019 (24.4)	1,414 (33.8)	409 (9.8)	4,182 (100.0)

## Discussion

The Rh blood group system exhibits complex genetic polymorphism and immunophenotypes, with significant variations in the geographic and ethnic distribution of the RhD-negative blood type. Among China's Han population, the proportion of RhD-negative individuals is approximately 0.3%, while among ethnic minorities, it ranges from 1% to 5% (7, 8). Therefore, in China, RhD-negative blood is also known as “Panda-Type Blood.” This decadal analysis reveals critical demographic anchors for optimizing RhD-negative donor recruitment in Chongqing. Understanding the demographic characteristics, donation behavior, and ABO/RhCcEe phenotype distribution of RhD-negative whole blood donors is essential for blood centers. This knowledge enables the development of targeted donor recruitment and retention strategies. Furthermore, it supports the establishment of a rare RhD-negative blood registry. Ultimately, these measures are crucial for ensuring the safety of clinical blood transfusion for RhD-negative patients.

Research consistently indicates that only a very small proportion of people donate blood regularly (9, 10). Many donors do not donate regularly and cease participating in voluntary blood donation after one or a few donations (11). A multi-center study in China revealed that first-time donors contributed 64% of the total blood collection volume, yet only 14% converted to repeat donors within 9 months (12). Although our study focused exclusively on RhD-negative donors, a similar pattern was observed. Analysis spanning a decade of RhD-negative blood donors in the Chongqing region demonstrated that the one-time donors constituted the largest proportion, accounting for 59.0% of all RhD-negative donors over this decade, while active donors represented the smallest group at only 7.4%. Attracting and retaining donors remains a primary challenge for blood services in the Chongqing.

One-time donors are predominantly comprised of students (33.2%), who constitutes a significant cohort of blood donors and serves as a reliable source for safe blood donation. University students typically maintain good physical health with high blood screening eligibility rates, while also demonstrating a strong sense of social responsibility (13, 14). This underscores the fact that campuses are high-yield recruitment grounds. However, the low retention rate of student in this study (evidenced by the fact that students accounted for only 9.6% of active donors) emphasizes the untapped potential of RhD-negative blood collection in higher education institutions. The low proportion of students among active donors (9.6%) must be interpreted considering that “student” is a transitional occupation. Our analysis, based on demographics at the first donation, identifies the student population as a primary entry point into the RhD-negative donor pool. The subsequent transition of these individuals into professional careers and older age groups likely facilitates their evolution into active donors, a dynamic process that should be investigated in future longitudinal studies. In order to convert this temporary group into committed donors, in the future, efforts should be made to develop more diverse RhD-negative blood donation recruitment campaigns in university, to continuously improve promotional strategies, and to focus on tapping the potential of the RhD-negative blood group of students donating blood on university campuses.

The age stratification further informs retention strategy. Active blood donors are predominantly comprised of older age groups (those aged 36–45 account for 28.9%; those aged 46 and above account for 26.4%), presenting a stark contrast to one-time donors (those aged 18–25 account for 49.2%). This suggests that publicity and recruitment for RhD-negative blood donation should employ precision targeting and enhanced appeal. This requires developing tailored approaches based on the key characteristics and interests of specific age groups. To effectively respond to the foreseeable blood supply challenges, a dual-strategy approach is essential. On one front, targeted recruitment should focus on attracting young donors to the RhD-negative blood donation program. This can be achieved by leveraging new media platforms for fashionable and interactive outreach, while simultaneously optimizing the public service platform for non-remunerated blood donation to enable seamless online booking, information queries, and electronic management. Concurrently, sustained efforts are needed to encourage both new and existing donors to maintain long-term donation commitment. Establishing personalized follow-up mechanisms and implementing intelligent, proactive appointment reminders will help foster a sense of belonging and honor among donors.

The phenotypic distribution, dominated by ccdee (50.7%) and Ccdee (36.9%), was largely consistent with reports from other regions in China, while also exhibiting unique local characteristics (15). Compared with other regions, the difference in the frequency of RhD-negative phenotypes was mainly manifested in ccdEe and CCdee. The frequency of ccdEe is higher than that of CCdee in regions such as Jinan, Beijing, and Hohhot, whereas in Nanchang, Liuzhou and other regions, the frequency of CCdee is higher than ccdEe, with obvious geographical differences between north and south (16–18). The Rh system antigens are highly immunogenic, readily stimulating the production of irregular antibodies through events like blood transfusion or pregnancy immunization (19, 20). In the study by Hui Ni et al., antibody-specific testing was conducted on 131 patients between April 2020 and January 2022. Among the 131 antibodies detected, 68 were Rh system antibodies, such as anti-E, anti-c, etc (21). Concurrently, in studies by Yuhong Zhao et al., antibodies detected unexpectedly during pre-transfusion screening were examined. It was found that among all 557 homologous antibodies, Rh blood group antibodies accounted for 57.99% (323/557), while anti-E antibodies constituted 82.35% (266/323) (22). Given that Rh antibodies are the most prevalent among clinically significant irregular antibodies, patients requiring multiple transfusion are at heightened risk of developing additional antibodies against other Rh antigens (e.g., anti-E, anti-c). Therefore, proactively identifying and cataloging the Rh phenotype (beyond just the D antigen) directly improves the efficiency of donor management and enhances clinical safety, especially for multi-transfused patients (23, 24). Firstly, in case RhD-negative patients develop other irregular antibodies to the Rh system, we can select RhD-negative donors purposefully, which improves the chances of cross-matching to ensure that the patients can obtain compatible RhD-negative blood in time. Secondly, and most critically for donor stewardship, this knowledge enables a data-driven, tiered retention strategy.

We recommend adopting a multi-tiered model for donor management. All RhD-negative donors are valued and engaged through standard community-building initiatives, while targeted retention programs are implemented for specific clinical needs. Donors with high-frequency, broadly compatible phenotypes like ccdee are prioritized as the backbone for routine inventory and are actively retained to ensure a stable supply for the broadest patient needs. Donors possessing very rare and immunogenic phenotypes (e.g., CCdEe, CcdEE) are enrolled in a specialized “rare case reserve” program. These donors will receive personalized communication and be contacted for targeted donations to meet specific, pre-identified patient needs in special circumstances. Meanwhile, given the low prevalence of RhD-negative individuals in China and the significant heterogeneity observed (with up to nine distinct phenotypes), the supply of this rare blood source is inherently limited and unevenly distributed across blood banks. This further underscores the necessity of establishing local RhD-negative donor registries and implementing cross-regional resource sharing.

By analyzing the gender, age, occupational distribution, and ABO/Rh antigen phenotype distribution among RhD-negative blood donors in Chongqing, our study provides scientific evidence for formulating precise and effective recruitment and retention strategies, offering data-driven insights to address practical challenges within the blood collection and supply sector. Notably, this decade-long analysis encompasses the global COVID-19 pandemic, a period that profoundly impacted blood services worldwide. Whilst the limited size of the donor cohort in this study precludes formal stratified analysis, the potential pandemic-related effects warrant careful consideration. Future longitudinal studies and regional comparisons will be crucial for comprehensively elucidating the specific quantitative and qualitative impacts of the pandemic on the recruitment and retention dynamics of rare blood donors.

## Conclusion

This decade-long study establishes key characteristics of RhD-negative blood donors in Chongqing: a donor pool reliant on one-time donors (59.0%), particularly students and young adults, with active donors being older and more highly educated. The phenotypic distribution is dominated by ccdee (50.7%) and Ccdee (36.9%), showing distinct regional variations. These findings highlight a critical need for differentiated management strategies: targeted youth recruitment and improved retention mechanisms to guide donors through life-stage transitions. Future studies should address the low conversion rate from one-time to regular donation.

## Data Availability

The raw data supporting the conclusions of this article will be made available by the authors, without undue reservation.
